# Navigating Culture and Crisis: Saudi Mothers’ Experiences of Family-Centered Care in Pediatric Intensive Care Units—A Qualitative Study

**DOI:** 10.3390/healthcare14101405

**Published:** 2026-05-20

**Authors:** Waleed M. Alshehri, Albandari Almutairi, Thurayya Eid, Asrar S. Almutairi, Rayhanah R. Almutairi, Bader M. Almutairy, Faihan F. Alshaibany, Wjdan A. Almutairi, Ashwaq A. Almutairi, Abdulaziz M. Alodhailah

**Affiliations:** 1 Department of Medical-Surgical Nursing, College of Nursing, King Saud University, Riyadh 11451, Saudi Arabia; 2 Department of Maternal and Child Health Nursing, College of Nursing, King Saud University, Riyadh 11451, Saudi Arabia; alafi@ksu.edu.sa; 3 Community and Psychiatric Mental Health Nursing Department, College of Nursing, Princess Nourah Bint Abdulrahman University, P.O. Box 84428, Riyadh 11671, Saudi Arabia; 4 Department of Community and Mental Health Nursing, College of Nursing, King Saud University, Riyadh 11451, Saudi Arabia; 5 Department of Nursing Administration and Education, College of Nursing, King Saud University, Riyadh 11451, Saudi Arabia; 6 College of Nursing, King Saud Bin Abdulaziz University for Health Sciences (KSAU-HS), Riyadh 11481, Saudi Arabia; 7 King Abdullah International Medical Research Center, Riyadh 11481, Saudi Arabia; 8 Ministry of National Guard Health Affairs, Riyadh 11426, Saudi Arabia; 9 Monash Nursing and Midwifery, Monash University, Melbourne, VIC 3800, Australia

**Keywords:** family-centered care, pediatric intensive care, qualitative descriptive, maternal experience, cultural brokering, Saudi Arabia, reflexive thematic analysis

## Abstract

**Background:** Family-centered care (FCC) is a foundational principle in pediatric healthcare, yet its implementation in culturally specific contexts remains poorly understood. In Saudi Arabia, Islamic values, collective family structures, and gendered caregiving norms shape how mothers engage with pediatric intensive care in ways that existing Western-derived FCC models do not fully capture. The aim of this study was to explore Saudi mothers’ experiences of family-centered care during their children’s pediatric intensive care unit (PICU) admissions, focusing on perceived barriers, cultural negotiations, and evolving advocacy strategies. **Methods:** A qualitative descriptive study was conducted with 17 Saudi mothers whose children had been admitted to PICUs across major hospitals in Saudi Arabia within the preceding 12 months. Semi-structured interviews lasting 40–70 min were conducted in Arabic using a pilot-tested, 15-item guide. Data were analyzed through Braun and Clarke’s six-phase reflexive thematic analysis. Trustworthiness was strengthened through member checking, reflexive journaling, negative case analysis, and investigator triangulation. Reporting adheres to the Consolidated Criteria for Reporting Qualitative Research (COREQ). **Result:** Five interconnected themes emerged: (1) confronting crisis and uncertainty, (2) renegotiating maternal identity, (3) brokering culture within biomedicine, (4) forging trust with care teams, and (5) evolving into advocates. These themes trace a developmental arc from initial disorientation through progressive empowerment, shaped at every stage by culturally grounded resources and constraints. Mothers functioned as cultural brokers performing invisible labor that healthcare systems neither recognized nor supported. **Conclusions:** Saudi mothers in PICUs engage in sophisticated cultural mediation between family systems and biomedical institutions under conditions of acute stress. Findings underscore the need for structurally embedded cultural responsiveness in PICU policy, including continuous cultural assessment, care-team continuity, and family advocacy support.

## 1. Introduction

### 1.1. Background and Significance

When a child is admitted to a pediatric intensive care unit (PICU), the entire family enters a world of technological complexity, clinical uncertainty, and emotional upheaval [1,2]. Family-centered care (FCC) positions families as essential collaborators rather than passive visitors and has become a guiding principle in pediatric healthcare [3]. Evidence consistently demonstrates that genuine family partnership improves clinical outcomes, reduces parental distress, and increases satisfaction for both families and providers [4,5,6]. Despite this evidence, translating FCC into everyday intensive care practice remains a persistent challenge, particularly where medical urgency, infection-control protocols, and space constraints compete with family presence [5].

The challenge deepens in culturally diverse settings, where values shape how families interpret illness, make decisions, and interact with providers [7]. In the Middle East, extended-family involvement in decisions, Islamic understandings of illness as part of divine decree, and gendered caregiver responsibilities all influence how FCC is received and enacted [8]. Saudi Arabia sits at a distinctive intersection of rapid healthcare modernization under Vision 2030, deeply rooted tribal and Islamic traditions, and an increasingly multinational healthcare workforce [9,10]. These forces create a unique set of tensions for family engagement in pediatric critical care [11].

Saudi mothers occupy a particularly pivotal position. Cultural norms designate them as primary caregivers and emotional anchors for sick children, yet the PICU may restrict their physical presence, subordinate maternal knowledge to clinical authority, and introduce male providers in settings where gender-segregated interaction is the cultural norm [12,13]. How mothers negotiate these tensions remains largely unexplored. Existing Saudi pediatric critical-care research has focused on clinical outcomes and provider perspectives, leaving maternal lived experience as a significant gap, specifically, no qualitative study has examined how Saudi mothers mediate between Islamic and extended-family values and the biomedical logic of PICU practice, nor how they enact the role of cultural broker within a system that does not formally recognize that labor. Cultural brokering, as used in this study, refers to the active mediation process whereby individuals translate between two different cultural systems, interpreting values, norms, and practices in both directions, to enable meaningful participation and communication. The present study addresses this gap by foregrounding maternal agency, cultural negotiation, and the FCC implications of both [14,15].

### 1.2. Aim

This study aimed to explore Saudi mothers’ experiences of FCC during their children’s PICU admissions, focusing on three dimensions: the emotional and relational challenges mothers faced, the cultural negotiations they engaged in, and the advocacy strategies they developed. The findings are intended to generate practice-relevant knowledge for building culturally responsive FCC models in Saudi and comparable healthcare contexts.

## 2. Methods

### 2.1. Design

A qualitative descriptive design, as articulated by Sandelowski [16,17] and characterized by Kim et al. [18], was selected for its capacity to produce a rich, data-near account grounded in participants’ own language and meaning-making. Unlike phenomenology or grounded theory, qualitative description stays close to the surface of data while still engaging interpretively with meanings and context. This orientation was well-suited to capturing the range and texture of maternal PICU experiences within a cultural context where prior qualitative evidence is sparse. Reporting follows the Consolidated Criteria for Reporting Qualitative Research (COREQ; [19]. As a qualitative study, this design does not aim for statistical generalizability; its purpose is analytic transferability—the production of theoretically grounded, contextually rich findings that practitioners and researchers can assess for applicability to their own settings. Accordingly, the study does not employ inferential statistics, and sample adequacy is evaluated through information power rather than sample-size formulas derived from quantitative traditions.

### 2.2. Setting and Participants

The study was conducted across PICUs in major hospitals in Saudi Arabia, spanning large tertiary centers in Riyadh and regional hospitals in smaller cities. Eligible participants were Saudi women aged 18 years or older whose child had been admitted to a PICU for at least 48 h within the preceding 12 months. The 48-h minimum ensured adequate exposure to FCC-relevant interactions; the 12-month window balanced recency of recall with emotional distance. Mothers whose children had died during or shortly after admission were excluded to minimize psychological harm, as were those with diagnosed psychiatric conditions that could impair participation.

### 2.3. Sampling and Data Adequacy

Participants were recruited through purposive sampling aimed at maximizing variation in maternal age, education, geographic region, child age, and length of stay. Initial recruitment occurred through professional networks associated with PICUs and social-media postings in parent-support groups. PICU nursing staff identified potentially eligible mothers and passed study information sheets to those who expressed interest; no staff member approached mothers during acute clinical crises or within the first 24 h of admission. Eligible mothers who expressed interest were contacted by a research team member not involved in their child’s clinical care. A potential selection bias should be acknowledged: mothers recruited through social-media parent-support groups may be more articulate, digitally connected, and comfortable with self-expression than those who did not engage with such networks. This may mean that mothers with the most negative or disempowering experiences—those least likely to seek peer community—are underrepresented, a limitation acknowledged in Section 5. Snowball sampling supplemented these efforts. Recruitment continued alongside ongoing analytical engagement until new interviews were deepening existing themes without generating new ones. This judgment was reached after the 15th interview; two additional interviews confirmed it. Consistent with Braun and Clarke’s [20] position that saturation is an inappropriate concept for reflexive thematic analysis, we assessed the dataset’s depth and breadth relative to the study aim, informed by the principles of information power [21]. The final sample comprised 17 mothers. In applying these principles, information power was judged adequate on the basis of: (a) a narrow, well-defined aim focused specifically on Saudi mothers’ PICU experiences; (b) a highly specific sample in which all participants shared the target experience; (c) engagement with established FCC and cultural brokering theory throughout data collection; (d) rich interview data averaging 48 min per participant; and (e) an inductive reflexive thematic analysis strategy that prioritizes interpretive depth. These five elements together justify a sample smaller than would be required for studies with broader or more heterogeneous aims.

### 2.4. Data Collection

Semi-structured interviews were conducted between 1 September and 31 December 2025 using a 15-item guide developed by the research team, reviewed by two external experts in pediatric nursing and qualitative methodology, and pilot-tested with two mothers not included in the final sample. The two reviewers held doctoral-level qualifications: one was a professor of pediatric nursing with expertise in Saudi critical-care contexts, and the other was a senior qualitative health researcher with experience in family-centered care studies in the Arab region. The guide was developed iteratively: initial items were generated from a synthesis of the FCC literature and the team’s clinical experience in Saudi PICU settings, then refined following expert review and pilot-testing. Pilot interviews led to clarification of three items and the addition of two probes targeting extended-family decision-making dynamics. The guide was organized around five domains: admission experiences, maternal roles and identity, barriers and challenges, healthcare-team interactions, and cultural and religious influences. Questions progressed from broad narrative prompts to focused probes.

All interviews were conducted face-to-face in Arabic by two team members, both native speakers trained in qualitative interviewing. Interview location was chosen by the participant. Interviews lasted 40–70 min (M = 48 min), were audio-recorded with consent, and were accompanied by field notes capturing contextual observations, nonverbal cues, and interviewer reflections. Emotional distress was monitored; breaks were offered and referral resources were available.

### 2.5. Researcher Reflexivity

The study was conducted by a multidisciplinary research team comprising nursing academics from Saudi Arabia and Australia. Reflexivity was integrated throughout the research process. The Saudi researchers brought contextual familiarity with local healthcare culture, language, and maternal health practices, while the international team member contributed an external lens that supported critical examination of culturally embedded assumptions. In terms of positionality, the Saudi team members are experienced nursing academics who have worked within or alongside PICU environments; several are mothers themselves, which enriched interpretive sensitivity but also required active monitoring of insider assumptions. The Australian team member had no prior professional ties to the participating institutions and served as an external auditor of interpretive claims. No team member had a prior personal relationship with any participant. These positional dynamics were documented in reflexive journals from the outset and surfaced explicitly in team analytical meetings to prevent unexamined cultural assumptions from shaping the analysis.

All researchers involved in analysis maintained reflexive notes during data collection and coding. These reflections were discussed in regular team meetings to identify and challenge assumptions, enhance analytic rigor, and strengthen the credibility and trustworthiness of the findings.

### 2.6. Data Analysis

Data were analyzed using Braun and Clarke’s [20] six-phase reflexive thematic analysis, conducted in Arabic to preserve cultural nuance before representative quotations were translated into English. Translation was verified through back-translation by an independent bilingual researcher.

Phase 1—Familiarization. Transcripts were read and re-read alongside audio recordings. Analytical memos documented initial impressions, emotional registers, and culturally specific references.

Phase 2—Coding. Inductive coding was conducted in NVivo 14. Two team members independently coded the first five transcripts; codes were compared, discussed, and reconciled before one coder continued while the other audited a random 30% subset.

Phase 3—Generating initial themes. Codes were clustered into candidate themes through collaborative mapping sessions involving four team members.

Phase 4—Reviewing themes. Candidate themes were reviewed against coded extracts and full transcripts. Two themes were merged, one was subdivided, and one was reabsorbed into subtheme-level analysis.

Phase 5—Defining and naming themes. Each theme received a concise name and a scope statement specifying its boundaries.

Phase 6—Writing up. The analytical narrative was drafted with quotations selected for representativeness and illustrative power.

### 2.7. Trustworthiness

Credibility was supported through prolonged engagement with transcripts, member checking with six participants who confirmed preliminary thematic descriptions, and investigator triangulation across a culturally diverse team. Dependability was enhanced through a documented audit trail of reflexive journals, meeting minutes, and progressive codebook versions. Confirmability was strengthened by negative case analysis, in which divergent accounts were actively sought and integrated. Transferability is supported by thick description of the setting, sample, and cultural context.

### 2.8. Ethical Considerations

Ethical approval was granted by the Institutional Review Board of King Saud University (IRB No. KSU-HE-25-955; approved 26 August 2025). All procedures complied with the Declaration of Helsinki [22]. Written informed consent was obtained following explanation of purpose, voluntary nature, confidentiality protections, and the right to withdraw. Pseudonym codes (M01–M17) replaced identifying information throughout. Readers should note that the prefix “M” in participant codes (e.g., M03, M11) denotes “Mother” and serves as a participant identifier; it is distinct from the statistical abbreviation “M” used in Table 1 to denote the arithmetic mean of continuous variables.

## 3. Findings

### 3.1. Participant Characteristics

Seventeen Saudi mothers participated. Their characteristics are summarized in Table 1.

The sample encompassed diversity in education, employment, geography, and child age and diagnosis, providing a broad experiential basis for the analysis. Hospital type was distributed as follows: tertiary government hospitals (*n* = 11), regional government hospitals (*n* = 4), and private hospitals (*n* = 2). The predominance of government hospital participants reflects the majority of PICU provision in Saudi Arabia and should be considered when assessing transferability to private-sector settings.

### 3.2. Thematic Overview

**Figure 1:**
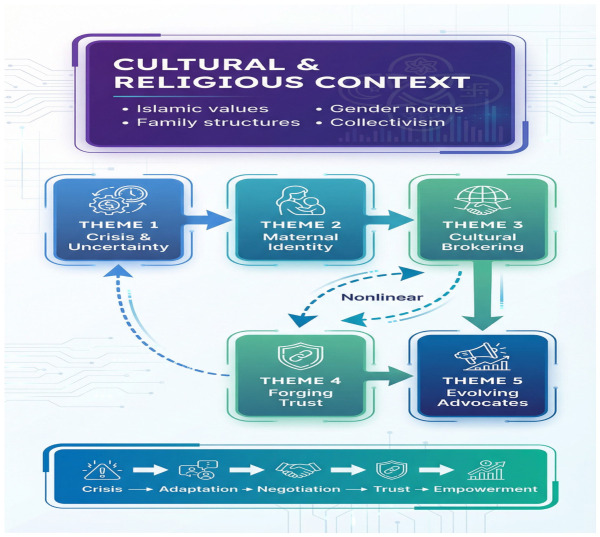
Thematic map illustrating the five interconnected themes emerging from Saudi mothers’ experiences of family-centered care in pediatric intensive care units (*n* = 17). The developmental arc progresses from initial crisis (Theme 1) through identity renegotiation (Theme 2), cultural brokering (Theme 3), trust-building (Theme 4), and advocacy (Theme 5). Dashed arrows indicate nonlinear movement, as clinical setbacks could return mothers to earlier experiential phases. The encompassing frame represents the cultural and religious context, including Islamic values, extended family structures, gender norms, and collective identity, that shaped all five themes. Subthemes are listed within each theme box.

Reflexive thematic analysis generated five interrelated themes tracing a developmental arc from initial crisis through progressive empowerment (Figure 1; Table 2).

### 3.3. Theme 1: Confronting Crisis and Uncertainty

Every participant recalled the PICU admission as a rupture in ordinary life. The sight of a critically ill child surrounded by ventilators, monitors, and intravenous lines overwhelmed mothers regardless of education level or prior hospital exposure. As M03 described:

“When I first saw my daughter in the intensive care unit, I couldn’t believe this was real. She looked so small surrounded by all those machines and tubes. I felt like I was in a nightmare.” (M03)

Other mothers echoed this sense of cognitive paralysis. M07 recalled that the medical team’s explanations became inaudible noise: “*The doctor kept talking about oxygen levels and medications, but nothing was going in. My mind just stopped”* (M07). M11 described a similar freeze: *“I could see their mouths moving, but all I could focus on was my son’s face and those tubes going into his body*” (M11).

The uncertainty was intensified by cultural expectations that mothers bear primary responsibility for their children’s welfare. M04 captured this burden: “*In our family, when your child is sick, everyone looks at you, the mother, first. I felt guilty for things I could not have controlled*” (M04). Religious faith emerged immediately as a coping resource. M02 noted: “*Before I even spoke to a doctor, I opened Quran on my phone. I needed God before I needed medicine*” (M02). M15 similarly described turning to collective family prayer: “*My mother and sisters came within the hour. We prayed together in the hallway because there was no room inside*” (M15).

A contrasting voice came from M14, whose second child had experienced a previous PICU admission. While she still felt fear, she described feeling somewhat better oriented: “*I knew what the monitors meant this time. I still cried, but I was not as lost as the first time*” (M14). This suggests that experiential familiarity may buffer, though not eliminate, the initial shock.

### 3.4. Theme 2: Renegotiating Maternal Identity

Once the initial shock began to subside, mothers confronted a deeper challenge: how to remain a mother when the most familiar expressions of mothering, holding, feeding, and bathing, were no longer possible. M09 articulated this tension:

“As a mother, I’m supposed to take care of my child when he’s sick. But in the intensive care unit, I couldn’t hold him or even touch him sometimes because of all the tubes. I had to find other ways to be his mother, talking to him, praying for him, making sure the nurses understood what he needed. It was the hardest thing I’ve ever done.” (M09)

M06 described standing outside the glass partition for hours: “*I would just watch him breathe. That was all I could do, watch and pray*” (M06). Several participants channeled their maternal energy into vigilant monitoring and information-gathering. M05, a university-educated mother, explained: “*I started reading everything I could about his condition on my phone. If I could not hold him, I could at least understand what was happening to him*” (M05). M12 focused on spiritual caregiving: “*I tied a small Quran verse to his bed. The nurse asked what it was, and when I explained, she smiled and left it there. That moment meant everything*” (M12).

The renegotiation was not linear. M16, whose child’s condition deteriorated unexpectedly after initial improvement, described a sharp regression: “*I thought we were getting better. Then his fever came back and the alarms started again. I was right back to that first night, helpless*” (M16). Over time, however, most mothers with longer stays reported arriving at a new maternal identity that combined traditional caregiving values with active clinical partnership. M08 reflected: “*By the third week, I knew every machine, every reading. I became part of the team in my own way*” (M08). This shift was echoed by others with extended stays. M02, whose son remained in the PICU for five weeks, described a similar transformation: “*I stopped being afraid of the doctors. I started preparing for rounds, writing my questions the night before. My husband said I had become like a nurse myself*” (M02). M14, who had a prior PICU experience with a different child, articulated the identity shift most explicitly: “*The first time I was in shock and helpless. This time I understood that my job was to be my child’s voice and her memory inside that unit. That is a real role, not less than the nurses*” (M14). Together, these accounts illustrate that identity reconstruction was not idiosyncratic but a recognizable trajectory among mothers whose stays exceeded two weeks.

### 3.5. Theme 3: Brokering Culture Within Biomedicine

Cultural negotiation permeated every interaction. Mothers served, often without institutional recognition, as cultural brokers translating between their family’s value system and the hospital’s biomedical logic.

Gender and modesty. Several mothers navigated interactions with male physicians within cultural parameters of modesty. M10 described: “*At first I did not feel comfortable speaking freely with the male doctor. But he was very respectful and always asked if I wanted a female nurse present. After a few days, I trusted him*” (M10). M17 offered a different perspective: “*I did not care about the gender of the doctor. I cared about who would save my son. When your child is dying, these things become less important*” (M17).

Religious integration. Prayer and Quranic recitation were not merely personal coping strategies but communal family practices requiring accommodation. M13 described the tension: “*Prayer time came five times a day, and we needed to pray. Sometimes it clashed with rounds or procedures. We learned to adjust; pray a bit early, pray a bit late; but it would have helped if the schedule had a space for it*” (M13). Not all experiences of religious accommodation were marked by tension. Some mothers recalled moments when the healthcare team actively integrated religious practice into daily care routines. M08 specifically credited her primary nurse’s awareness: “*She knew our prayer times without being asked. She would finish any non-urgent procedure before the call to prayer and step back. It was a small thing for her, but it showed us she saw us as a whole family, not just a medical case*” (M08). M12, who had attached a Quranic verse to her son’s bed, described a similarly affirming interaction: the nurse not only left the verse in place but asked its meaning, an act that M12 described as transforming a tense relationship into one of trust. These accounts suggest that even modest gestures of accommodation by staff can shift the quality of the care partnership substantially.

Extended-family involvement. M01 captured the complexity of collective decision-making:

“In our culture, when a child is sick, the whole family is involved. My husband’s mother wanted to be part of every decision, and my brothers had opinions about which doctors we should see. The medical team seemed confused about who they should talk to. We had to explain our family structure and find ways to include everyone while still getting the medical care our child needed.” (M01)

Not all mothers found extended-family involvement burdensome. M03 described her family as her greatest resource: “My sisters organized a schedule: one always stayed with me so I was never alone. They brought food, they brought comfort. Without them, I would have collapsed” (M03).

The labor of cultural brokering fell almost entirely on mothers, adding an invisible layer of cognitive and emotional work to an already overwhelming situation.

### 3.6. Theme 4: Forging Trust with Care Teams

Trust was not assumed; it was earned through observed competence and demonstrated cultural respect. Mothers described a two-stage assessment in which they first evaluated whether a provider appeared clinically skilled and then gauged whether that provider acknowledged family values. M08 illustrated this:

“The best nurses were the ones who learned our names and remembered what was important to our family. They would ask about our other children, respect our prayer times, and explain everything they were doing with our son. When we had different nurses every day, it felt like starting over each time.” (M08)

M04 recalled a positive exemplar: “There was one nurse from the Philippines. She did not speak Arabic, but she learned three words: ‘Alhamdulillah,’ ‘inshallah,’ and my daughter’s name. That small effort made me trust her completely” (M04). M11 described the opposite experience: “One doctor spoke to my husband but not to me, even though I was the one who stayed with our son every day. I felt invisible” (M11).

Language barriers compounded the challenge. Non-Arabic-speaking expatriate providers, while often technically proficient, sometimes struggled to convey empathy in culturally resonant terms. M15 recalled: “*The translator was not always available. Once I had to use my phone to translate what the doctor was saying. Important information should not depend on Google Translate*” (M15).

Consistency of staff assignment emerged as a powerful moderator. Mothers who experienced frequent rotations reported repeated labor of re-explaining cultural needs, whereas those with consistent teams described faster trust development and greater peace of mind.

### 3.7. Theme 5: Evolving into Advocates

As familiarity with the PICU grew, mothers transitioned from passive recipients of information to active agents who questioned, negotiated, and coordinated care. M10 described the underlying motivation:

“I learned that I had to speak up for my daughter because she couldn’t speak for herself. I had to make sure the doctors understood not just her medical needs but also what would comfort her and help her heal. I had to explain why certain things were important to our family. It was exhausting, but it was my responsibility as her mother.” (M10)

The advocacy trajectory followed a learning curve. M07 described her progression: “In the first days, I was afraid to ask anything. By the second week, I was writing down questions before rounds. By the end, I was requesting meetings with the team” (M07). M13 described a pivotal turning point: “When I noticed that the medication schedule had changed without anyone telling me, I went straight to the charge nurse. Before this experience, I would never have done that” (M13). M05 similarly described how persistent information-seeking evolved into confident advocacy: “By the fourth week I was not asking for permission to know things about my son. I was asking because I had a right to know. That shift—from asking permission to claiming a right, was the biggest change in me” (M05). M09, initially overwhelmed by the clinical environment, connected her advocacy growth directly to FCC values: “They told us this was family-centered care, which meant we were partners. Once I understood that, I felt I had a reason to speak. Not just as a worried mother but as a member of the team” (M09). These accounts illuminate the conditions under which formal FCC framing became a resource mothers could draw on to legitimize their participation.

Cultural advocacy was a distinctive dimension: mothers invested time in educating providers about religious practices, dietary requirements, and family decision-making structures, labor that amounted to informal cross-cultural training. Several mothers with extended stays became informal mentors. M16 described helping a newly admitted family: “*A young mother came in looking terrified; just like I had been. I sat with her, told her what to expect, showed her where to pray. I wished someone had done that for me*” (M16).

### 3.8. Thematic Integration

The five themes are not discrete stages but interlocking dimensions of a single adaptive process. Confronting crisis (Theme 1) generated the emotional urgency that drove identity renegotiation (Theme 2). As mothers found new ways to enact care, they were better positioned to navigate cultural negotiations (Theme 3), which facilitated trust-building (Theme 4) and ultimately enabled effective advocacy (Theme 5). The trajectory was nonlinear; clinical setbacks could propel a mother from confident advocacy back to crisis mode within hours. Religious and family resources served as stabilizing forces throughout.

## 4. Discussion

### 4.1. Culturally Embedded Maternal Adaptation

The central finding of this study is that Saudi mothers’ PICU experiences constitute culturally embedded adaptive processes unfolding across emotional, relational, and structural dimensions simultaneously. The initial crisis responses, including shock, cognitive freeze, and existential fear, resonate with findings from Western PICU studies [6,23], confirming the universality of parental distress in pediatric critical care. What distinguishes the present findings is the cultural specificity of both the stressors and the resources. The expectation that mothers bear primary responsibility for a child’s welfare intensified guilt when outcomes were uncertain, while Islamic beliefs about divine decree and communal prayer provided coping mechanisms that occupy a more peripheral and differently configured role in Western FCC literature. Spirituality and religion do appear in Western critical care research—notably in studies of palliative care, end-of-life decision-making, and family coping [see, e.g., Davidson et al. [5]]—but they function primarily as individual psychological resources rather than as communal, structurally embedded obligations with scheduling and gender implications. In the Saudi context, collective prayer practice, mandatory across five daily times, Islamic restrictions on physical contact with non-mahram providers, and the interpretive frame of illness as divine decree constitute institutional-level demands on FCC delivery that Western models have not specifically operationalized [8].

The identity renegotiation captured in Theme 2 extends work by Heydarpour et al. [24] on maternal role adaptation in neonatal intensive care by revealing an additional cultural layer. Saudi mothers were not simply adjusting to a medical environment; they were renegotiating their social identity within a system that defines good motherhood through physical presence and hands-on care. The creative redirections they found, such as spiritual caregiving, vigilant monitoring, and informational mastery, represent culturally patterned forms of agency rather than generic coping.

### 4.2. Cultural Brokering as Invisible Labor

Perhaps the most distinctive contribution is the identification of mothers as cultural brokers performing bidirectional translation between family systems and biomedical institutions. While cultural brokering has been applied in migration health [25], its application to domestic cultural dynamics within a rapidly modernizing healthcare system is novel. Mothers translated medical information into culturally meaningful terms for extended family members while translating family values into requests that the healthcare system could process. This labor was cognitively demanding, emotionally exhausting, and institutionally invisible.

The asymmetry of cultural adaptation is a critical finding. Mothers overwhelmingly reported adjusting their practices to fit hospital norms, while experiencing far less reciprocal accommodation from healthcare systems. This imbalance echoes broader critiques of cultural competence frameworks that burden non-dominant groups with the work of adaptation [15] and suggests that FCC models developed in Western, individualist contexts may carry implicit assumptions about family structure, decision-making, and religious practice that do not map onto Saudi realities. Specifically, mainstream FCC frameworks tend to assume a nuclear family unit as the primary decision-making entity, position the mother or primary carer as the single point of contact with the clinical team, treat religious observance as a personal and time-flexible practice, and presuppose gender-neutral clinical interactions. None of these assumptions hold uniformly in the Saudi PICU context studied here. Extended-family decision-making structures required the team to negotiate with multiple stakeholders; prayer obligations imposed non-negotiable scheduling constraints; gender norms shaped the maternal comfort with male providers. These findings suggest that FCC implementation in Saudi and comparable Gulf-state PICUs requires not simply the addition of cultural sensitivity training but a structural reconfiguration of how partnership, presence, and participation are defined within the model. Maternal spiritual and religious resources, including Quranic recitation, communal supplication, and Islamic acceptance of illness as divine will, did not merely buffer distress; they shaped the mothers’ expectations of what culturally competent FCC should look like, including whether the care team acknowledged these practices as integral to care rather than peripheral to it.

### 4.3. Communication, Continuity, and Trust

The trust-building processes described by participants highlight the interplay between clinical competence and cultural sensitivity. Mothers’ two-stage assessment, namely first competence and then cultural respect, suggests that sensitivity alone is insufficient without perceived clinical excellence. This finding aligns with Saifan et al. [14], who identified both structural and cultural barriers to nurse–family ICU communication, and extends it by demonstrating how trust erodes when care-team continuity is disrupted. Every staff rotation forced mothers to restart the cultural-explanation cycle, producing fatigue and, in some cases, withdrawal from active engagement.

The challenges mothers faced with non-Arabic-speaking expatriate providers underscore the need for language support that extends beyond translation to include cultural interpretation. This is a distinction the healthcare system does not currently make. In a workforce where a substantial proportion of clinicians are recruited internationally, structured cultural orientation and integrated mediation services would address a structural deficit rather than an individual failing. From an FCC perspective, the implications are direct. The care partnership at the heart of FCC depends on mutual intelligibility—not only linguistic but cultural. When communication is limited to clinical transactions, families perceive providers primarily as technical operators rather than partners, which reduces parental involvement and erodes the relational foundation on which FCC rests [3,5]. The mother who relies on a smartphone translation application to understand a physician’s explanation of her child’s prognosis is not a partner in care; she is a passive recipient struggling to bridge a structural gap. Every staff rotation that forces a mother to re-explain prayer times, dietary restrictions, or family decision-making hierarchies adds cognitive load that diminishes her capacity to participate in care discussions as an equal stakeholder. These are FCC implementation failures, not communication failures attributable to individuals.

### 4.4. Maternal Advocacy as Emergent Competence

The evolution from passive distress to active advocacy echoes findings from Karakul et al. [23] on parental advocacy in pediatric palliative care but adds a distinctively cultural dimension. Saudi mothers advocated not only for clinical quality but for cultural and religious accommodation, a dual mandate that required them to balance cultural deference to medical authority with the equally cultural imperative to protect their child. The emergent peer-mentoring behavior among mothers with longer stays suggests that family-to-family support could be institutionalized through structured navigator programs adapted to the Saudi context. Connecting this to FCC principles is important: peer navigation aligns directly with FCC’s recognition of families as partners not only in their own child’s care but also in supporting other families within the unit. Experienced Saudi mothers who had already mapped the cultural terrain of the PICU—knowing prayer spaces, understanding how to request gender-concordant providers, learning how to integrate extended family into care discussions—were performing exactly the kind of knowledge-brokering that formal FCC family liaison programs aim to provide. Institutionalizing this through trained family navigator roles would both validate the informal labor mothers already perform and embed it within a structure that makes it available to all newly admitted families rather than only those fortunate enough to encounter a long-staying peer.

### 4.5. Implications for Practice and Policy

These findings carry concrete implications for PICU care delivery. Cultural assessment should shift from a one-time admission checklist to an ongoing, dynamic process—for example, a brief structured cultural conversation at 48 h, one week, and fortnightly thereafter, covering prayer scheduling preferences, gender-concordance needs, family communication preferences, and dietary requirements. Care-team continuity should be prioritized as a recognized mechanism for reducing family burden—concretely, this could mean primary-nurse assignment for the duration of a PICU admission and a documented “family cultural profile” embedded in the electronic record and reviewed at each handover. Cultural competence training should move beyond awareness to scenario-based skill development drawn from real maternal narratives—the narratives documented in this study offer a ready source of teaching cases for such training. Hospitals should establish formal cultural mediation roles—distinct from interpreting services—staffed by bilingual professionals with training in both clinical communication and Saudi sociocultural norms. The cultural mediator role would serve as the institutional analogue of what mothers are currently doing informally and unpaid, and review institutional policies on visiting, prayer accommodation, dietary provision, and family meetings through the lens of the negotiations mothers described [26].

## 5. Strengths and Limitations

This study offers one of the first in-depth qualitative examinations of Saudi maternal experience in pediatric intensive care, generating culturally grounded findings from a diverse sample spanning multiple hospitals, geographic regions, and family circumstances. The use of Braun and Clarke’s reflexive thematic analysis provided a rigorous and epistemologically coherent analytical framework, and the culturally diverse research team brought both insider understanding and outsider critical scrutiny to interpretation. Conducting and coding interviews in Arabic preserved cultural nuances that are often lost in translation-dependent designs. Reporting adheres to the 32-item COREQ checklist.

Several limitations should be acknowledged. The study captured only the maternal perspective; fathers, children where developmentally appropriate, and healthcare providers would add essential viewpoints. Voluntary participation may have attracted mothers more comfortable articulating their experiences, potentially underrepresenting those with the most negative encounters. Social desirability bias cannot be excluded, as cultural norms valuing respect for authority may have moderated criticism of providers. The retrospective design introduces the possibility of recall reconstruction; prospective, longitudinal data collection would yield complementary evidence. The exclusion of bereaved mothers was a deliberate a priori exclusion criterion—specified in the protocol and approved by the IRB—adopted to minimize the risk of re-traumatization in a population already navigating profound grief. Bereaved mothers were therefore not inadvertently omitted; their exclusion reflects an ethical judgment that the potential harm of participation outweighed the research benefit at this stage. However, the substantive cost of this decision should not be underestimated. Mothers whose children died during or shortly after PICU admission may have had qualitatively different experiences of FCC: their encounters with care-team communication, decision-making support, spiritual accommodation, and cultural responsiveness at end-of-life carry particular weight for FCC policy. Future research, designed with appropriate bereavement-sensitive protocols, ethics safeguards, and peer support provisions, should specifically include this population. Their voices are not merely additive; they are essential to understanding FCC in its most consequential moments. Finally, although core themes may resonate across Gulf-state and Middle Eastern contexts, cultural specificity limits direct transferability beyond the Saudi setting.

## 6. Recommendations

Healthcare providers should integrate ongoing cultural assessment into routine PICU care planning, moving beyond a single admission inquiry to revisit families’ evolving cultural and religious needs throughout the stay. Staffing models should prioritize care-team continuity, and where full continuity is impractical, structured handover tools that capture family-specific cultural information should be used. Cultural competence training for all PICU staff should incorporate simulation-based learning and case scenarios drawn from real maternal narratives, embedded in both pre-service curricula and continuing professional development. Hospitals should establish formal cultural mediation services and peer-support programs connecting experienced families with newly admitted ones. Institutional policies on visiting hours, prayer accommodation, dietary provisions, and family meetings should be audited and revised in light of the cultural negotiations documented here. Future research should adopt multi-perspective designs including fathers and providers, test the effectiveness of culturally responsive FCC interventions, and explore long-term family outcomes beyond PICU discharge.

## 7. Conclusions

This study reveals that Saudi mothers whose children are admitted to PICUs do far more than wait at the bedside. They confront crisis, reconstruct their maternal identity, broker between their family’s cultural world and the hospital’s biomedical logic, build trust one interaction at a time, and grow into skilled advocates. At the center of this process lies invisible cultural labor, including bidirectional translation, emotional management, and religious adaptation, that current systems neither recognize nor support. Culturally responsive FCC cannot be achieved through surface accommodations; it requires structural change that embeds cultural responsiveness into institutional fabric. When healthcare systems meet families where they are, honoring cultural identity as a resource rather than an obstacle, the partnership at the heart of family-centered care becomes genuine.

## Figures and Tables

**Table 1:** Participant Characteristics (*n* = 17).

Characteristic	Detail
Maternal age (years)	Range: 23–45; M = 32.4, SD = 6.2
Child’s age at PICU admission	Range: 2 months–14 years
Length of PICU stay	Range: 3 days–8 weeks
Maternal education	Primary (*n* = 4); Secondary (*n* = 7); University (*n* = 5); Postgraduate (*n* = 1)
Employment status	Homemaker (*n* = 9); Part-time (*n* = 4); Full-time (*n* = 3); Student (*n* = 1)
Geographic setting	Major urban centers and smaller cities across Saudi Arabia
Marital status	All married, living with spouse

**Table 2:** Themes, Subthemes, and Descriptions.

Theme	Subthemes	Description
1. Confronting crisis and uncertainty	Shock and disbelief; fear for survival; incomprehension of medical complexity; environmental overwhelm; persistent prognostic anxiety	The abrupt transition to the PICU precipitates emotional disorientation compounded by the unfamiliar technological environment
2. Renegotiating maternal identity	Loss of caregiving control; deference to clinical authority; creative expressions of mothering; preserving emotional bonds; gradual identity reconstruction	Mothers redefine how they enact care when physical caregiving is restricted
3. Brokering culture within biomedicine	Gender-appropriate interactions; integrating religious practice; managing extended-family involvement; serving as cultural translator; adapting traditions to medical constraints	Mothers mediate between their family’s cultural world and the hospital’s biomedical logic
4. Forging trust with care teams	Assessing competence; evaluating cultural sensitivity; valuing continuity; communicating cultural needs; managing language barriers	Trust develops incrementally through perceived clinical skill and cultural respect
5. Evolving into advocates	Information-seeking; assertive communication; cultural education of providers; care coordination; healthcare navigation skills	Mothers progressively acquire skills to represent their child’s medical and cultural needs

## Data Availability

All data supporting the findings are included in the article. Full interview transcripts are not publicly available due to participant confidentiality but can be requested from the corresponding author with ethics approval.
